# ZHX2 drives cell growth and migration via activating MEK/ERK signal and induces Sunitinib resistance by regulating the autophagy in clear cell Renal Cell Carcinoma

**DOI:** 10.1038/s41419-020-2541-x

**Published:** 2020-05-07

**Authors:** Liangsong Zhu, Rong Ding, Hao Yan, Jin Zhang, Zongming Lin

**Affiliations:** 1 Department of Urology, Zhongshan Hospital, Fudan University, Shanghai, China; 2 Department of Obstetrics and Gynecology, International Peace Maternity and Child Health Hospital, School of Medicine, Shanghai Jiao Tong University, Shanghai, China; 3 Department of Urology, Ren Ji Hospital, School of Medicine, Shanghai Jiaotong University, Shanghai, China

**Keywords:** Renal cell carcinoma, Molecular biology

## Abstract

Zinc fingers and homeoboxes 2 (ZHX2) was found as a novel VHL substrate target, and acted as an oncogenic driver in ccRCC. However, the detailed mechanism of ZHX2 in ccRCC development remains elusive, and no research has focused on studying ZHX2 in drug resistance yet. A tissue microarray with 358 ccRCC samples was used to determine the expression of ZHX2 in ccRCC patients. VHL-deficient cell line 786-O and VHL-normal cell line CAKI-1 was used for lineage reprogramming by transfecting with lentivirus. The in vitro and in vivo experiments were performed with these new cell lines to determine the mechanism of ZHX2 in ccRCC development and drug resistance. Immunohistochemistry analysis showed that ZHX2 was not highly expressed in ccRCC tumor tissues, only 33.2% (119/358) patients have high ZHX2 expression. However, high ZHX2 was significantly associated with advanced Fuhrman grade (*p* = 0.004), and proved to be an independent prognosis factor for progression-free survival (*p* = 0.0003), while there is no significant correlation with overall survival. We further discovered that ZHX2 overexpression could increase VEGF secretion and transcriptional activate the MEK/ERK1/2 and promote its downstream targets. We also found ZHX2 overexpression induce Sunitinib resistance though activating autophagy and the combination treatment of Sunitinib and Chloroquine could significantly rescue the phenomenon. In summary, these results indicate that ZHX2 drivers cell growth, migration though increase VEGF expression, and transcriptional activate MEK/ERK1/2 signaling pathway, and could induce Sunitinib resistance by regulating self-protective autophagy, these may provide new insight in advanced ccRCC treatment.

## Introduction

Since the tumor suppressor gene *VHL* was identified as the key point of the Von Hippel-Lindau (VHL) disease in 1993^1^, it has been clearly proved that hypoxia or gene mutation could lead to the inactivation of VHL and induce the loss function of VHL complex (VBC, including elongin B and C), which targeting hypoxia-inducible factors (HIFs) for ubiquitylation and proteasomal degradation^2–5^. As well studied, large scale of chromatin immunoprecipitation sequencing (Chromatin immunoprecipitation (ChIP)-seq) showed that over 800 genes could directly regulated by HIFs, such as vascular endothelial growth factor (VEGF)^6,7^, and HIFs could also regulate downstream gene expression by trans activating gene encoding microRNAs and enzymes in chromatin modification^8–10^. VHL-HIFs signal is a important role in the development of clear cell renal cell carcinoma (ccRCC), and the tyrosine kinase inhibitors (TKIs) that target the VHL substrate HIF signal have showed treating benefit in patients with advanced ccRCC. However, drug resistance still occurs during following treatment in most patients, seems identify additional VHL substrates is urgent to improve therapeutic outcome of ccRCC patients^11^. Qing Zhang et al.^12^ have found that Zinc fingers and homeoboxes 2 (ZHX2) is a novel VHL substrate transcription factor by using a genome-wide human cDNA library strategy; and they reported that depletion of ZHX2 could inhibit the proliferation of *VHL*-deficient ccRCC cell lines through impairing NF-κB pathway. Though, the detailed mechanism of ZHX2 in ccRCC is still not clear, and whether it plays a role in TKIs drug resistance is also unknown.

ZHX2 is a member of ZHX protein family which including ZHX1 and ZHX3, and these proteins contains two zinc-fingers and four or five homeodomains. Several studies report that ZHX proteins are ubiquitously expressed and primary found in the nucleus and functions as transcriptional repressors^13–15^. However, there were some conflicting data when deep investigating the detail function of ZHX2 in human malignant cancers. Lv et al.^16^ demonstrated that ZHX2 promoter region was hypermethylated in hepatocellular carcinoma (HCC) samples, suggesting it might functions as a tumor suppressor. On the other side, another immunohistochemical research reported that ZHX2 was highly expressed in tumor samples compared to adjacent tissues in HCC patients, and the prognostic analysis showed ZHX2 was associated with poorer outcomes^17^, seems it act as an oncogenic biomarker.

As renal cancer, the evidence accumulating from past decade suggests that the HIF2a is the key factor for progression, and the HIF1a might function as a tumor suppressor^18,19^. These phenomena are very different from other vascularized tumors. However, HIF2a inhibitor has limited treating response in ccRCC patients, so there must be some kind of extra signals to promote cancer proliferation^20^. ZHX2 as a novel VHL substrate transcription factor like HIF2a, there may be synergy function between two signaling pathways, and this may be responsible for TKI drug resistance. In this study, we further investigate the ZHX2 expression in ccRCC patients, and explore the detailed mechanism about ccRCC progression in ZHX2 reprogramming cell lines. Further, we try to find out whether it is related with sunitinib resistance.

## Materials and methods

### Tissue immunohistochemistry

Tissue microarrays (TMAs) that contain 358 ccRCC samples (including tumor and adjacent tissues) were kindly provided by Prof. Jin Zhang. The pathologic information of all samples were determined by two experienced pathologists, and the comprehensive clinicopathologic data included gender, age, tumor size, the TNM stage, and Fuhrman grade were determined by according to the standard classification systems^21,22^. Annual laboratory test and MRI or CT scan was used to detect metastasis during the follow-up examination. Deep investigation of the ZHX2 localization and protein expression were preformed by immunohistochemistry (IHC) analysis according to the standard streptavidin-peroxidase method (Zymed Laboratories Inc, San Francisco, CA, USA). The primary antibody against ZHX2 (Rabbit ZHX2 antibody, 112232, Genetex, CA, USA) was diluted into 1:50 and PBS was as negative control. The localization and expression of ZHX2 were assessed independently by two observers (Dr Zhu and Dr Ding). The expression level of ZHX2 was divided into two groups according to the IHC score, which was calculated by staining percentage×intensity as our previous study^23^. This study was approved by the Ethical Review Boards of Zhong Shan Hospital Fudan University.

### Cell lines with Lentiviral transduction and siRNA transfection

The two ccRCC cell lines used in our study were 786-O and CAKI-1 (Purchased from American Type Culture Collection, ATCC, Manassas, VA, USA). 786-O was VHL-deficient cells while CAKI-1 was VHL-normal cells^24^. All cell lines were maintained in the recommended medium 1640 supplemented with 10% heat-inactivated fetal bovine serum (FBS, Gibco, Australia), 1% GlutaMAX, 1% nonessential amino acids, and 1% sodium pyruvate. Cultures were maintained at 37 °C with 5% CO2, and the medium was changed at least twice weekly.

The lentivirus expression human ZHX2 (LV-*ZHX2*), lentivirus silence VHL expression (LV-sh*VHL*), and the control lentivirus (LV-NC) were purchased from GENECHEM GROUP (shanghai, China). LV-*ZHX2* was used to transduce 786-O to increase ZHX2 expression, LV-sh*VHL* and LV-*ZHX2* were used to transduce CAKI-1 for lineage reprogramming. New stable cell lines were established and used in further research after cultivating in the medium containing puromycin (5 μg/mL, Sigma) for 7 days (change the medium every there days). The protein and RNA level of ZHX2 were tested by western blot and quantitative real-time PCR respectively (Detail information of lentivirus was collected in Supplementary Table S1).

The ZHX2, EPAS1 (HIF-2a) and respective negative control small interfering RNAs (siRNAs) (Purchased from GenePharma Company, shanghai China.) were transfected with lipofectamine RNAiMAX reagent (Invitrogen, Carlsbad, CA, USA) following the manufacturer’s instructions.

### Cell proliferation and drug experiments

The proliferation ability of new reprogrammed 786-O and CAKI-1 cell lines were determined by Sulforhodamine B (SRB) assay in 96-well plates according to the manufacture’s instruction. What’s more, the cells also plated into 6-well plates (1.5 × 10^5^ cell/well) and incubated overnight at 37 °C, then cell counts by using electronic cell counter (Invitrogen) were taken every 24 h for 96 h post incubation for another verification.

Sunitinib and Chloroquine (CQ) were purchased from Selleck (Shanghai, China.), and dissolved in nuclease-free water. Sunitinib applied to cell lines at a final concentration of 5 μM/L, while CQ applied at 10 μM/L. The inhibition effect of Sunitinib and the combination effects of Sunitinib + CQ were determined by cell count ratios with a time manner.

### Enzyme-linked immunosorbent assay

The cells were seeded in 6-well plates with 2 × 10^5^ cells/well for 24 h, and change the medium with 2 ml serum-free medium for another 24 h. Then cell supernatant was collected and centrifuged to remove the debris. The human VEGF ProQuantum Immunoassay Kit (A35602 ThermoFisher, USA) was used to measure the secreted VEGF.

### Wound healing assay

ccRCC cells were seeded in 6-well plate with 5 × 10^5^ cell/well and cultured until the cells were fully grown. Then scraped the cells in a straight line by using a 200 μl pipette tip to make a scratch, images were captured after washing with PBS twice and replaced with serum-free medium at 0 h, 24 h, and 48 h.

### Transwell migration assay

The new reprogramming ccRCC cell lines were seeded in the transwell inserts (pore size: 8.0 μm; Corning, Lowell, MA, USA) respectively with appropriate number of cells (3 × 10^4^ cells/well). The serum-free medium was in the upper migration stoppers while medium with 10% FBS serum was in the bottom wells. After 24 h, the invaded cells of each cell lines on the lower side of membrane were fixed in 95% methanol and stained with crystal violet according to the manufacture’s instructions.

### Western blot analysis

Western blot procedure was performed according to the standard protocol and the protein lysates were obtained from cultured cells with different treatment. Proteins were separated by 10% SDS-PAGE and then transferred onto a nitrocellulose membrane (Millipore, Temecula, CA, USA). After blocking with non-fat milk for nearly 1 h at room temperature, the membranes were incubated overnight at 4 °C with following primary antibodies: ZHX2 (Genetex, CA, USA), ERK1/2, p-ERK, LC3, AKT, p-AKT, STAT3, p-STAT3, JNK, and GAPDH (Cell Signaling Technology, Boston, MA, USA). Then the membranes were washed three times and incubated with secondary antibody at room temperature for 2 h. Then immunoreactive bands were detected by using ECL system (Beyotime, Jiangsu, China) according to the manufacture’s instruction, and the GAPDH were used as a loading control. The Image J 1.47V software (http://imagej.nih.gov/ij) was used for semi-quantification of the western blot experiments.

### Flow cytometry for cell apoptosis

For cell apoptosis analysis, cells that harvested after siRNA interference assay were collected according to the standard protocol (FITC Apoptosis Detection Kit). The apoptosis cells were measured by FACS Calibur flow cytometer (Becton-Dickinson, Mountain View, CA). All experiments were repeated three times.

### Quantitative real-time PCR

The QRT-PCR was performed according to the standard protocol, and the human β-actin was used as normalized control and each measurement was performed in triplicate. The primer sequences are presented in Supplementary Table S2.

### Chromatin immunoprecipitation and luciferase reporter assay

Chromatin immunoprecipitation for ZHX2 was performed according to the manufacture’s protocol (SimpleChIP Plus Enzymatic Chromatin IP Kit9005, CST, Boston, Massachusetts, USA), and the binding enrichment of MEK1 (also named MAP2K1) promoter (Forward primer: 5′-ccctagattcctttgtgctgc-3′; Reverse primer: 5′-cggttcgggtcgaaggaa-3′) was normalized against that in the input samples. The ZHX2 binding site of MAP2K1 promoter was clone into luciferase reporter pGL3-MAP2K1, and pGL3-basic as negative control. The luciferase activity was determined by using a Dual-Luciferase Assay Kit (E1910, Promega Biotechnology, USA) according to its manufacturer’s instructions.

### Autophagy protein labeling experiment

RFP-LC3 single-label-adenovirus was purchased from HanBio Company (Shanghai China). The LV-NC and LV-ZHX2 786-O cells were transfected with RFP-LC3 adenovirus, and drug experiments performed after its stable expression. RFP intensity was observed under fluorescence microscope after 24 h treatment. Autophagy intensity was determined by counting the number of red fluorescence.

### In vivo animal experiments

All animal experiments procedures were approved by the Animal Care and Use Committee of Zhongshan Hospital Fudan University (Shanghai, China). For subcutaneous tumor implantation, an equal number of 786-O cells with LV-ZHX2 or LV-NC (2 × 10^6^ cells) were resuspended in PBS and matrigel (BD, USA) at 1:1 ratio. Then injected into flank region of the six-week-old female athymic nude mice. Tumor growth was monitored and measured twice a week, and tumor weight was measured in the end of in vivo experiments. The samples were collected and saved in 4% paraformaldehyde for further Ki-67 staining.

### Statistical analysis

Univariate and multivariate analysis were used to determine the independent prognosis factors. Overall survival and Progression-free survival curves were plotted by Kaplan–Meier method and compared with the log-rank test. Significant difference between different treatments groups were tested by using a two-tailed, paired *t*-test analysis (Graphpad Software, Inc., CA, USA). All *P* values of < 0.05 were assigned significance.

## Results

### Overexpression of ZHX2 predicts worse outcome in ccRCC patients

**Fig. 1:**
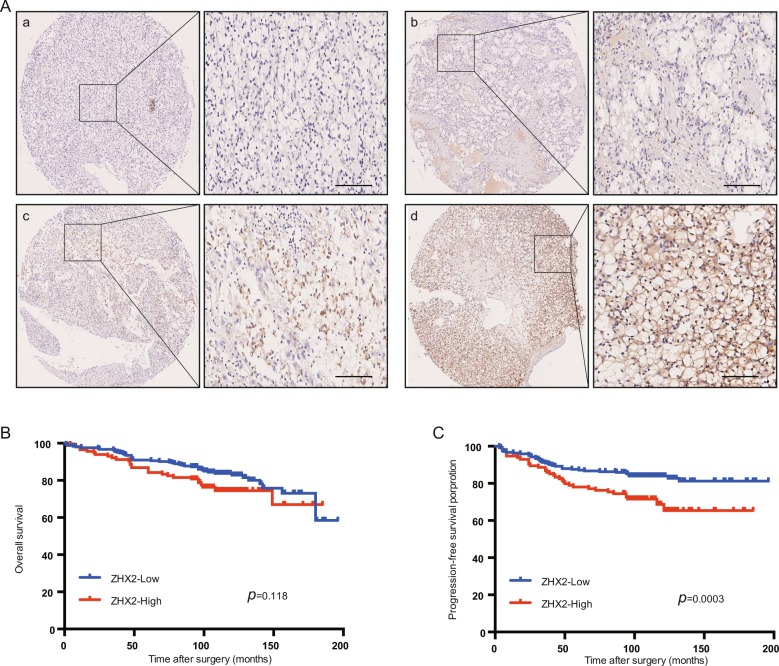
Overexpressed ZHX2 in ccRCC patients is correlated with worse clinical outcome. **a** The representative IHC images of ZHX2 protein expression level in ccRCC samples. a, negative; b, low; c, moderate; d, strong. Bar 100μm. **b** The association between different ZHX2 expression level and OS in ccRCC patients. Kaplan–Meier and log-rank test analysis were used to compare the two groups (*p* = 0.118). **c** The association between ZHX2 expression level and PFS in ccRCC patients. Kaplan–Meier and log-rank test analysis were used to compare the two groups (*p* = 0.0003).

The protein expression of ZHX2 was detected by IHC in 358 ccRCC patients, the characteristic information was showed in Table 1. As showed in Fig. 1a, ZHX2 was mainly localized in nucleus and we divided the patients into two groups according to the ZHX2 expression level (Fig. 1a, b represent low expression group while c, d represent high group). Only 33.2% (119/358) patients showed a high expression of ZHX2 (Table 1), but high expression of ZHX2 was significantly associated with advanced Fuhrman grade (*p* = 0.004) and metastasis after surgery (*p* = 0.009). The prognostic analysis showed that the patients with high ZHX2 have worse progression-free survival (PFS) while there was no significant relation with overall survival (OS) (Fig. 1b, c). We also searched the OS data in TCGA database and the result showed no correlation as well (Supplementary Fig. S1a). Univarivate and multivariate Cox regression analysis showed the higher expression of ZHX2 had independent predictive value for PFS in ccRCC patients. (HR = 0.567, *p* = 0.035, Supplementary Table S3–S4), which means ZHX2 might act as a tumor promoter that contributed to occurrence and recurrence of ccRCC.

**Table 1:** The ZHX2 expression and clinicopathological characteristics in ccRCC patients.

	Patients		Tumoral ZHX2 expression		
Characteristics	*n*	%	Low	High	*P*-value
All patients	358	100	239	119	
Gender					0.548
Male	254	70.9	172	82	
Female	104	29.1	67	37	
Age(years)					0.970
≤55	178	49.7	119	59	
>55	180	50.3	120	60	
TNM stage					0.215
I + II	341	95.3	230	111	
III + IV	17	4.7	9	8	
pT stage					0.436
T1 + T2	344	96.1	231	113	
T3 + T4	14	3.9	8	6	
pN stage					0.150
N0	349	97.5	235	114	
N1	9	2.5	4	5	
pM stage					0.080
M0	352	98.3	237	115	
M1	6	1.7	2	4	
Fuhrman grade					0.004*
I + II	297	83	208	89	
III + IV	61	17	31	30	
Tumor size(cm)					0.278
≤4	186	52	129	57	
>5	172	48	110	62	
Metastasis after surgery					0.009*
Yes	74	20.7	40	34	
No	284	79.3	199	85	

*Means significant difference.

### ZHX2 is involved in ccRCC cell proliferation and migration

**Fig. 2:**
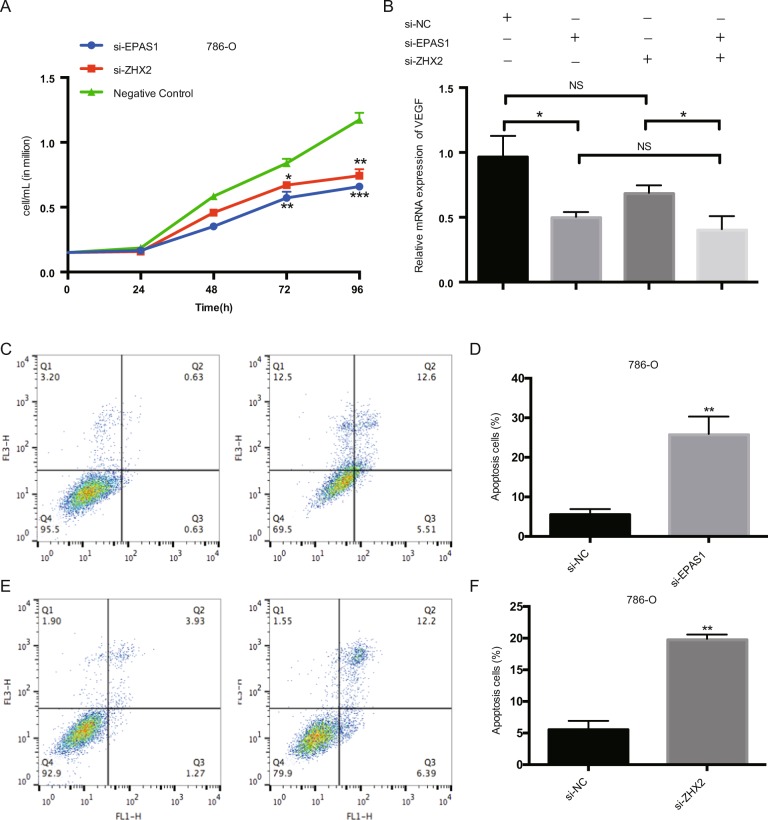
ZHX2 koncking down inhibits growth of 786-O cells. **a** The proliferation assay showed that ZHX2 knockdown significantly decreased cell proliferation in 786-O cells as well as EPAS1 in 72h and 96h. **b** The mRNA level of VEFGA in 786-O cells after ZHX2 or EPAS1 knocking down (tested in 72h). **c**, **d** Flow cytometry showed that the cell apoptosis ratio was significantly increased after ZHX2 or EPAS1 knockdown. All experiments were repeated double times or more. *p < 0.05, **p < 0.01 and ***p < 0.001.

As reported, ZHX2 depletion could inhibit the VHL-deficient ccRCC cell growth. We used the siRNA to knockdown the ZHX2 expression in 786-O cells as well as EPAS1 (Encode HIF-2a), and both genes silenced could inhibit the cell proliferation (Fig. 2a). We also detected the VEGF expression, one of the most important downstream targets of VHL-HIF signal, and the VEGF level was significantly decreased in si-EPAS1 cells while no significant reduction was observed in si-ZHX2 cells (Fig. 2b). Apoptosis analysis showed that either EPAS1 or ZHX2 knockdown could induce cell apoptosis (Fig. 2c–f), and wound healing assay showed both can inhibit cell migration (Supplementary Fig. S1b, c), which means that ZHX2 may have similar function with HIF-2a in ccRCC proliferation.

**Fig. 3:**
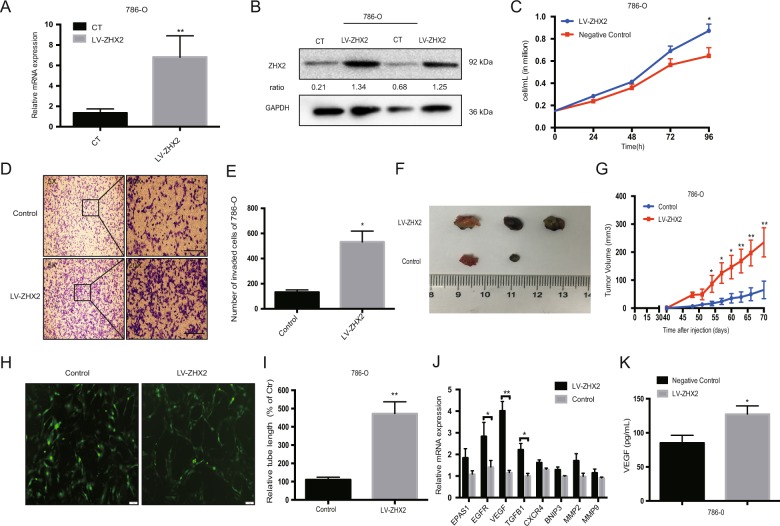
Elevated ZHX2 expression promotes the proliferation and migration of VHL-deficient ccRCC cells. **a**, **b** The mRNA and protein expression of ZHX2 after LV-ZHX2 transfection in 786-O cell line. The ratio in western blot semi-quantification was normalized by GAPDH respectively. **c** The proliferation assay showed ZHX2 overexpression promoted 786-O cells proliferation with a time manner. **d**, **e** The migration ability was significantly increased in ZHX2 overexpression cells compare with negative control in 786-O cells. Bar 100μm. **f**, **g** The xenograft tumor model of two reprogrammed 786-O cell lines, and tumor volume was much bigger in LV-ZHX2 group. **h**, **i** Tube formation could be seen in LV-ZHX2 cells under fluorescence microscope. The quantitative measurement showed the reprogrammed 786-O with ZHX2 overexpression had more tube length than negative control (*p* < 0.01). **j** The mRNA expression of some HIFs downstream genes in 786-O with LV-ZHX2 cells and negative control. **k** ELASA assay showed ZHX2 overexpression could increase VEGF secretion in 786-O cells. All experiments were repeated double times or more. *p < 0.05, **p < 0.01 and ***p < 0.001.

**Fig. 4:**
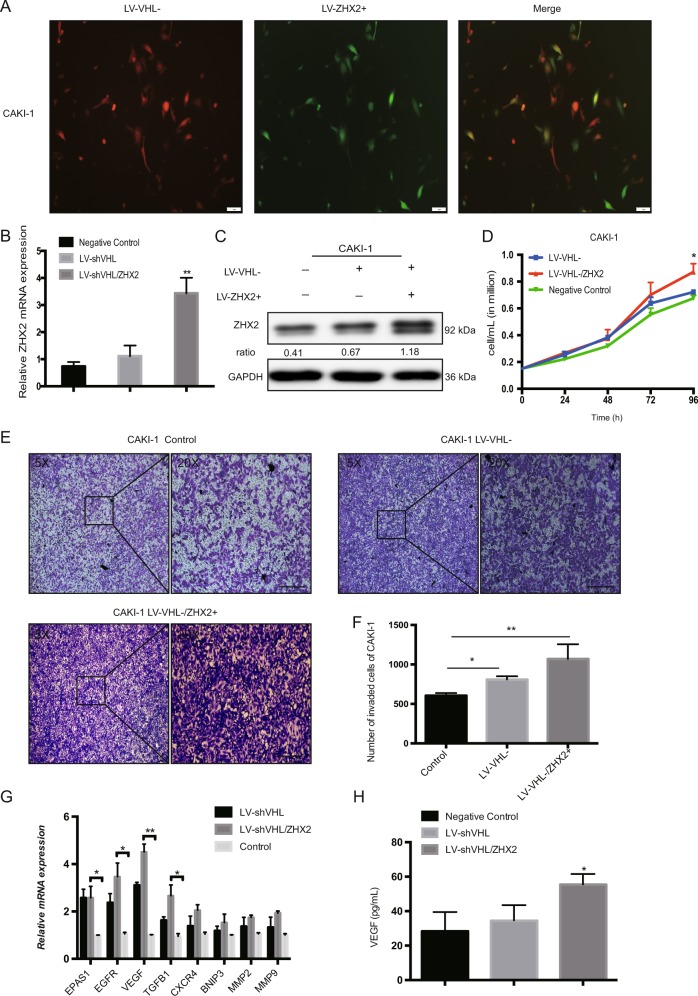
VHL silence and ZHX2 overexpression increase the cell proliferation and migration in VHL-normal ccRCC cells. **a** The representative images CAKI-1 after lentivirus-shVHL and LV-ZHX2 transfection. sh-VHL in red, LV-ZHX2 in green, and double reprogrammed in yellow. **b**, **c** The mRNA and protein expression of ZHX2 in reprogrammed CAKI-1 cells respectively. The ratio in western blot semi-quantification was normalized by GAPDH respectively. **d** The proliferation assay showed ZHX2 overexpression could promote cell growth after 96h in CAKI-1 cells. **e**, **f** The migration ability was also increased in new CAKI-1 cells with VHL silencing and ZHX2 overexpression. Bar 100μm. **g** The mRNA expression of some HIFs downstream genes in LV-shVHL/ZHX2 group and other groups in new CAKI-1 cells. **h** ELASA assay showed reprogrammed CAKI-1 could increase VEGF secretion as well. All experiments were repeated three times. *p < 0.05, **p < 0.01 and ***p < 0.001.

Further, we used lentivirus LV-ZHX2 to overexpress ZHX2 expression in 786-O cells (VHL-deficient RCC cell line). Both mRNA and protein level of ZHX2 were effectively up-regulated in new cell line (Fig. 3a, b). Proliferation assay showed 786-O with LV-ZHX2 could significantly promote cell growth after 96 h compared with negative control (Fig. 3c). Wound healing assay and Transwell assay also showed ZHX2 overexpression could increase the migration ability in 786-O cells compared with control group (Fig. 3d, e, Supplementary Fig. S2a, b). Furthermore, the xenograft tumor models were established by using 786-O with LV-ZHX2 and LV-NC respectively, as showed in Fig. 3f, average tumor volume was much bigger in LV-ZHX2 group (*p* < 0.01, Fig. 3g), and the tumor weight was showed heavier in LV-ZHX2 group (*p* < 0.01, Supplementary Fig. S2c) either. Besides, we performed IHC staining of Ki-67 in mouse tumors as well (Supplementary Fig. S3). These results indicated that the ZHX2 had strong promotive effect on cell proliferation and migration. Interestingly, tube formation could be seen under fluorescence microscope in 786-O/LV-ZHX2 cells (Fig. 3h, i), and the immunofluorescence staining of CD31 was more expressed in reprogrammed 786-O cells (Supplementary Fig. S4). We also detected some HIF’s downstream targets and found the mRNA expression of EGFR, VEGF, and TGFB1 were significantly increased compared with control group (Fig. 3j). Enzyme-linked immunosorbent assay (ELISA) assay was used to measure the secreted VEGF level in different cell lines, and ZHX2 overexpression cells may secret more VEGF to promote its self-tube formation and proliferation (Fig. 3k). At the same time, we used lentivirus silence VHL expression (LV-sh*VHL*) and LV-ZHX2 to reprogram the CAKI-1 cells (VHL-normal RCC cell line) (Fig. 4a). VHL silence and ZHX2 overexpression in CAKI-1 could increase cell proliferation and migration as well (Fig. 4b–f, Supplementary Fig. S2c, d). The mRNA expression of EGFR, VEGF, and TGFB1 were up-reguated in reprogram CAKI-1 cells as well as the secret VEGF (Fig. 4g, h). These phenomena are consistent with the previous results that ZHX2 and HIFs may have some same mechanism in the process of promoting cancer.

### ZHX2 promote ccRCC growth by transcriptional activate the MEK1/ERK1/2 signaling pathway

**Fig. 5:**
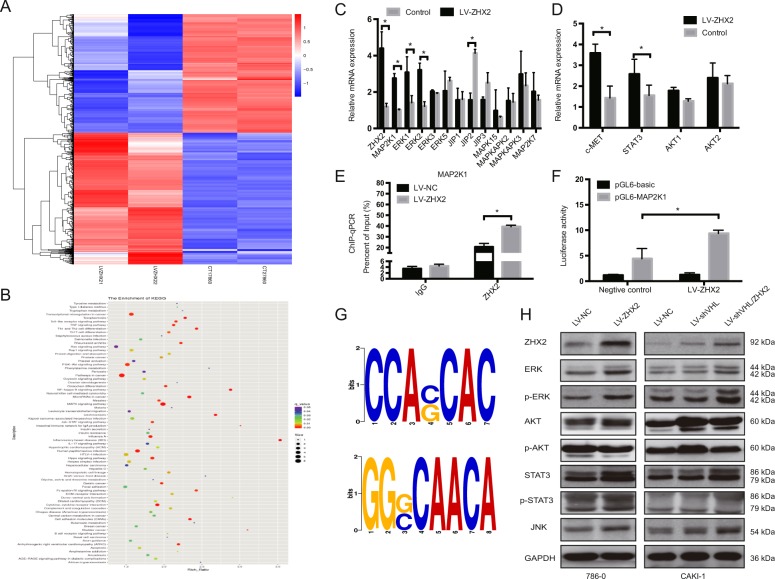
ZHX2 promotes ccRCC growth by transcriptional activates the MEK1/ERK1/2 signaling pathway. **a** The heat map of total differential genes in 786-O cells with LV-ZHX2 and negative control. **b** KEGG enrichment analysis was performed to explore the related pathways according to the RNA-seq data. **c**, **d** The mRNA expressions of related downstream genes in MAPK/ERK1/2 pathway in 786-O/LV-ZHX2 cells. **e**, **f** The ChIP-qpcr and luciferase assays were showed ZHX2 could direct bind to the promoter of MAP2K1 in 786-O cells. **g** The predicted binding motif of ZHX2 in genome. **h** The western blot assay was used to test the protein level of MEK-ERK signal in reprogrammed 786-O and CAKI-1 cells. All experiments were repeated double times. *p < 0.05, **p < 0.01, and ***p < 0.001.

To identify the more detail mechanism of ZHX2 in ccRCC cell proliferation and migration, RNA-sequence analysis was performed. As demonstrated in Fig. 5a, the heat-map showed the overall change of mRNA expression pattern, the upregulated genes indicated in red while downregulated genes in green. The enrichment of KEGG analysis showed several cancer-related pathways were activated in ZHX2 overexpression cells, especially MAPK signaling pathway (Fig. 5b). Then we detected the specific genes’ mRNA expression in MAPK pathway, and results showed MAP2K1, ERK1, and ERK2 were significantly increased in 786-O/LV-ZHX2 cells compared with negative control. Also the downstream genes of ERK pathway that c-MET and STAT3 were upreguated as well (Fig. 5c, d). These results suggest that MAP2K1-ERK1/2 pathway was transcriptionally upregulated upon ZHX2 overexpression. What’s more, we used ChIP-qPCR to determine whether ZHX2 could specifically bind to the promoter of MAP2K1 (which encode MEK1 protein and the direct upstream of ERK). The ChIP and Luciferase assays showed ZHX2 as a transcription factor can specifically target to the promoter of MAP2K1 (Fig. 5e, f). In the meantime, the ChIP-sequence also predicted the binging motifs of ZHX2 on genome (Fig. 5g). We examined the protein level of ERK pathway later, ERK with its phosphorylation and downstream STAT3 with its phosphorylation were increased as well as JNK, and no obvious change was showed in AKT (Fig. 5h), and the semi-quantification of the western blot were showed in Supplementary Fig. S5a. Similar, we test the MAPK-related protein expression in mouse tissue as well (Supplementary Fig. S5b). Taking together, our findings indicated that ZHX2 could direct activate the MEK1-ERK1/2 pathway in ccRCC cell lines.

### ZHX2 could induce sunitinib resistance by activating autophagy

**Fig. 6:**
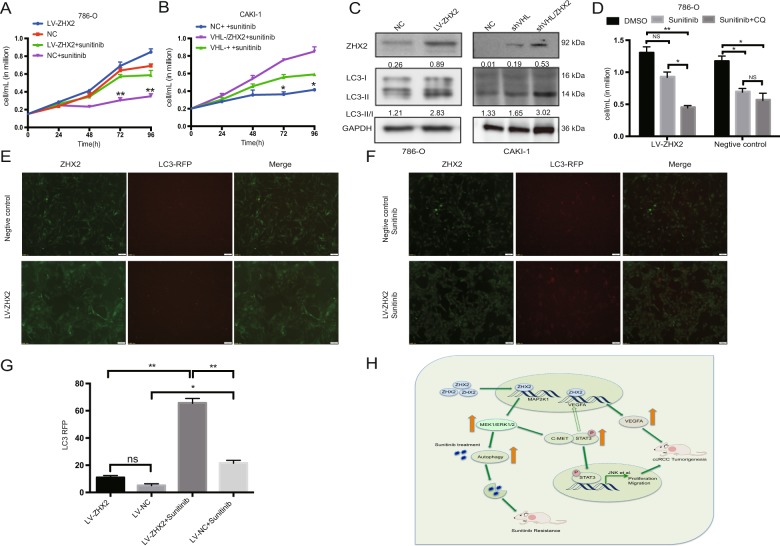
ZHX2 could induce Sunitinib resistance by activating autophagy. **a**, **b** The sunitinib treatment experiments in new reprogrammed 786-O and CAKI-1 respectively. **c** The autophagy related proteins LC3-I/II were tested by western blot in new reprogrammed 786-O and CAKI-1 respectively. The ratio in western blot semi-quantification was normalized by GAPDH respectively. **d** The combination treatment of sunitinib and CQ could significantly increase the inhibition rate in 786-O/LV-ZHX2 cells. **e**–**g** The single-label-adenovirus RFP-LC3 was used to detect the LC3 expression before and after sunitinib treatment respectively. The quantitative measurements of LC3 were performed under fluorescence microscope. **h** All experiments summarized in a schematic diagram. The solid arrow is the conclusion of this study, while the hollow arrow is the experimental guess. All experiments were repeated double times. *p < 0.05, **p < 0.01, ***p < 0.001, ns not significant.

As previous work has reported, MAPK-ERK pathway may be involved in tyrosine kinase drugs resistance^25,26^. Then we tested the inhibition rate of sunitinib in reprogramed ccRCC cell lines (Supplementary Fig. S6a), and choose 5 μmol/L for further investigation. As showed in Fig. 6a, b, the inhibitory effect of sunitinib was significantly decreased in the cell lines with overexpression of ZHX2. In this process, we found that many vesicles were turned up after treating with sunitinib in LV-ZHX2 cells, while there was no such change in control group (Supplementary Fig. S6c). So we wondered if autophagy was involved in this situation, and autophagy related proteins LC3-I/II were tested after sunitinib treatment in cell lines by western blot. The ratio of LC3-II/LC3-I was increased in ZHX2 overexpression cells, which meant autophagy was over-activated (Fig. 6c). The combination treatment of sunitinib and autophagy inhibitor CQ could significantly increase the inhibition rate in LV-ZHX2 cells (Fig. 6d). For further verification, we used RFP-LC3 single-label-adenovirus to detect the expression of LC3 after sunitinib treatment. Compared with the control group, the adenovirus labeled red light was significantly increased in LV-ZHX2 cells (Fig. 6e, g), although sunitinib treatment can also increase LC3 expression in normal ccRCC cells. Therefore, we put forward the assumption that ZHX2 overexpression could activate the MEK-ERK signaling pathway and reduce the inhibition effect of sunitinib by activating self-protective autophagy. So we proposed the following schematic diagram to help illustrate these mechanisms in which ZHX2 participates in ccRCC development (Fig. 6h) and ultimately serves as a potential therapeutic target.

## Discussion

ZHX2 was found as a novel VHL substrate transcription factor, and VHL could regulate its protein stability when it’s hydroxylated, just like HIFs. Although previous research has well illustrated VHL in the regulation of ZHX2 in ccRCC’s development, little is known about the downstream signaling pathway of ZHX2 as a transcription factor. In this study, we focused on the biological function of ZHX2 in ccRCC and the potential molecular downstream targets. Firstly, we tested the localization and expression of ZHX2 and only 33.2% of ccRCC patients have high expression in nuclear. We observed that high ZHX2 expression significantly correlated with worse outcome, and ZHX2 could activate VEGFA like HIF-2a did, and it markedly promoted cell proliferation, migration by transcriptional activating MEK1/ERK1/2 signaling. In this process, the expression of downstream MEK1 target genes, such as ERK1/2, STAT3, and JNK were upregulated, which may responsible for the tumor angiogenesis and metastasis of ccRCC.

The role of ZHX2 in different tumorigenesis is controversial. Kawata et al.^13^ reported it worked as a transcriptional repressor, and the changes of ZHX2 could regulate several oncogenes expression in human disease^16^. Similarly, Nagel et al.^15^ demonstrated some mechanisms that chromosomal rearrangement are related to decrease the expression of ZHX2 in Hodgkin lymphoma, including enhancing binding sites inactivation, upstream genes MSX1 and XBP1 reduction, and MSX1-corepressor H1C overexpression. In summary, their research proved that ZHX2 was involved in this regulative network and functioned as a tumor suppressor in B-cells malignant tumor as well. Yue et al.^27^ also discovered that ZHX2 inhibited the growth of HCC cell lines by transcriptional represses of two key cell cycle regulators, Cyclin A and Cyclin E^27^. Later, Wu et al.^28^ reported ZHX2 could protect hepatocytes from lipid deposition disorder in non-alcoholic fatty liver disease to retard cell growth and its-related HCC progression. All these researches illustrated that in the process of disease and tumorigenesis, ZHX2 usually played the role of tumor suppressor gene.

Nevertheless, Zhang et al.^12^ proposed that ZHX2 was a novel VHL substrate transcription factor and functioned as an oncogenic driver in the development of ccRCC. In his research, higher ZHX2 was found to express in most of ccRCC samples as well as HIF2a than those of the paired normal tissues. Depletion of ZHX2 could significantly inhibit ccRCC proliferation in vitro and in vivo. Here, we found that ZHX2 knocking down by siRNA could increase cell apoptosis and inhibit cell migration as well. Further, we used lentivirus to overexpress the ZHX2 in ccRCC cell lines to investigate the detail mechanism. Our study demonstrated that ZHX2 had some same functions as HIFs, and can activate VEGF signal. More importantly, RNA-seq analysis showed that ZHX2 could activate some other signals, especially MAPK/ERK pathway. ERK1/2 is a subfamily of MAPK signaling pathway, and it is regarded as a vital role to promoter cell survival and proliferation by entering into the nucleus after phosphorylation^29^. STAT3 is one of the important downstream molecules in ERK pathway, activated ERK1/2 can enhance transcription activity by regulating the phosphorylation of STAT3 in nucleus^30,31^. Our result showed that the ZHX2 overexpression increased the phosphorylation of ERK while both STAT3 and phosphorylated STAT3 were increased as well. STAT3 also has been recognized as a critical factor in cell growth and metastasis of various human malignant tumors^32,33^. The studies show that VEGF is the key factor of tumor angiogenesis, which is involved in the formation of tumor blood vessels and provides conditions for tumor invasion and metastasis. STAT3 is proved to be one of the regulatory factors of VEGF and it can mediate VEGF expression in two different ways. On the one hand, activated STAT3 can promote the transcription of HIF and regulate the expression of VEGF^34^; on the other hand, activated STAT3 can promote the transcription activity of VEGF by directly binding to its promoter^35^. In our in vitro study, ZHX2 silencing could part decrease VEGF expression and ZHX2 overexpression could significantly activate VEGF. The result may be because ZHX2 is upstream of VEGF or ZHX2 regulate VEGF through MEK/ERK/STAT3 signal.

Sunitinib is still one of the standard treatments in advanced ccRCC patients, researchers searched the GEO database and microarray data in sunitinib-resistant RCC tumors and demonstrated that MAPK/ERK pathway was excessive activating in sunitinib-resistance RCC tumors^25^. The mechanism of sunitinib resistance is still unknown, as a new transcription factor of VHL, ZHX2 may regulate VEGF signal and activate MAPK/ERK signal pathway, so we speculate that ZHX2 may be related to sunitinib resistance. Sunitinib inhibition test confirmed that the inhibition effect of sunitinib on the ccRCC with high expression of ZHX2 was obviously decreased and this result can be rescued by combination treatment with CQ. Meanwhile, RFP-LC3 single-label-adenovirus was used to detect autophagy related protein expression. These results indicated that ZHX2 overexpression might induce sunitinib resistance by self-protective autophagy, and the more detail mechanism investigation is still needed.

In summary, we test and verify the role of ZHX2 in ccRCC development, and ZHX2 facilitates the proliferation and migration in ccRCC cell lines by activating the MEK1/ERK1/2 signaling pathway. Our study also provides a new perspective on the involvement of ZHX2 in sunitinib resistance. These discoveries may give a new insight for ccRCC treatment.

## Supplementary information

Supplementary Table

supplementary figure legend

supplementary S1

supplementary S2

supplementary S3

supplementary S4

supplementary S5

supplementary S6
