# Correction: ZHX2 drives cell growth and migration via activating MEK/ERK signal and induces Sunitinib resistance by regulating the autophagy in clear cell Renal Cell Carcinoma

**DOI:** 10.1038/s41419-021-03678-9

**Published:** 2021-04-14

**Authors:** Liangsong Zhu, Rong Ding, Hao Yan, Jin Zhang, Zongming Lin

**Affiliations:** 1 Department of Urology, Zhongshan Hospital, Fudan University, Shanghai, China; 2 Department of Obstetrics and Gynecology, International Peace Maternity and Child Health Hospital, School of Medicine, Shanghai Jiao Tong University, Shanghai, China; 3 Department of Urology, Ren Ji Hospital, School of Medicine, Shanghai Jiaotong University, Shanghai, China

**Keywords:** Renal cell carcinoma, Molecular biology

Correction to: *Cell Death and Disease*

10.1038/s41419-020-2541-x published online 07 May 2020

The original version of this article unfortunately contained a mistake in Fig. 5. The correct figure can be found below. The authors apologize for the error.

**Fig. 5:**
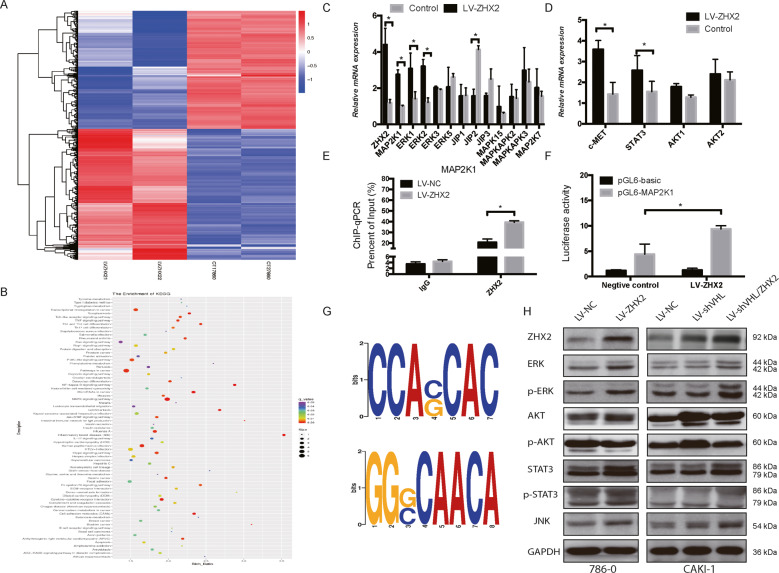
ZHX2 promotes ccRCC growth by transcriptional activates the MEK1/ERK1/2 signaling pathway. **a** The heat map of total differentialgenes in 786-O cells with LV-ZHX2 and negative control. **b** KEGG enrichment analysis was performed to explore the related pathways according tothe RNA-seq data. **c**, **d** The mRNA expressions of related downstream genes in MAPK/ERK1/2 pathway in 786-O/LV-ZHX2 cells. **e**, **f** The ChIP-qpcr andluciferase assays were showed ZHX2 could direct bind to the promoter of MAP2K1 in 786-O cells. **g** The predicted binding motif of ZHX2 in genome. **h** The western blot assay was used to test the protein level of MEK-ERK signal in reprogrammed 786-O and CAKI-1 cells. All experiments wererepeated double times. *p < 0.05, **p < 0.01, and ***p < 0.001.

