# Supplementary material for: ZHX2 drives cell growth and migration via activating MEK/ERK signal and induces Sunitinib resistance by regulating the autophagy in clear cell Renal Cell Carcinoma
Source: Cell Death Dis. 2020 May 7;11(5):337. doi: 10.1038/s41419-020-2541-x (PMC7206010; doi:10.1038/s41419-020-2541-x)
Supplement: Supplementary file 1 — Supplementary Table [file 41419_2020_2541_MOESM1_ESM.docx]

Supplementary

**Table S1** The information of LV-ZHX2 and LV-shVHL lentivirus.

**LV-ZHX2**

CCGTTTTTGGCTTTTTTGTTAGACGAAGCTTGGGCTGCAGGTCGACTCTAGAGGATCCCCGGGTACCGGTCGCCACCATGGCTAGCAAACGAAAATCTACAACTCCATGCATGGTTCGGACATCACAAGTAGTAGAACAAGATGTGCCCGAGGAAGTAGACAGGGCCAAAGAGAAAGGAATCGGCACACCACAGCCTGACGTGGCCAAGGACAGTTGGGCAGCAGAACTTGAAAACTCTTCCAAAGAAAACGAAGTGATAGAGGTGAAATCTATGGGGGAAAGCCAGTCCAAAAAACTCCAAGGTGGTTATGAGTGCAAATACTGCCCCTACTCCACGCAAAACCTGAACGAGTTCACGGAGCATGTCGACATGCAGCATCCCAACGTGATTCTCAACCCCCTCTACGTGTGTGCAGAATGTAACTTCACAACCAAAAAGTACGACTCCCTATCCGACCACAACTCCAAGTTCCATCCCGGGGAGGCCAACTTCAAGCTGAAGTTAATTAAACGCAATAATCAAACTGTCTTGGAACAGTCCATCGAAACCACCAACCATGTCGTGTCCATCACCACCAGTGGCCCTGGAACTGGTGACAGTGATTCTGGGATCTCGGTGAGTAAAACCCCCATCATGAAGCCTGGAAAACCAAAAGCGGATGCCAAGAAGGTGCCCAAGAAGCCCGAGGAGATCACCCCCGAGAACCACGTGGAAGGGACCGCCCGCCTGGTGACAGACACAGCTGAGATCCTCTCGAGACTCGGCGGGGTGGAGCTCCTCCAAGACACATTAGGACACGTCATGCCTTCTGTACAGCTGCCACCAAATATCAACCTTGTGCCCAAGGTCCCTGTCCCACTAAATACTACCAAATACAACTCTGCCCTGGATACAAATGCCACGATGATCAACTCTTTCAACAAGTTTCCTTACCCGACCCAGGCTGAGTTGTCCTGGCTGACAGCTGCCTCCAAACACCCAGAGGAGCACATCAGAATCTGGTTTGCCACCCAGCGCTTAAAGCATGGCATCAGCTGGTCCCCAGAAGAGGTGGAGGAGGCCCGGAAGAAGATGTTCAACGGCACCATCCAGTCAGTACCCCCGACCATCACTGTGCTGCCCGCCCAGTTGGCCCCCACAAAGGTGACGCAGCCCATCCTCCAGACGGCTCTACCGTGCCAGATCCTCGGCCAGACTAGCCTGGTGCTGACTCAGGTGACCAGCGGGTCAACAACCGTCTCTTGCTCCCCCATCACACTTGCCGTGGCAGGAGTCACCAACCATGGCCAGAAGAGACCCTTGGTGACTCCCCAAGCTGCCCCCGAACCCAAGCGTCCACACATCGCTCAGGTGCCAGAGCCCCCACCCAAGGTGGCCAACCCCCCGCTCACACCAGCCAGTGACCGCAAGAAGACAAAGGAGCAGATAGCACATCTCAAGGCCAGCTTTCTCCAGAGCCAGTTCCCTGACGATGCCGAGGTTTACCGGCTCATCGAGGTGACTGGCCTTGCCAGGAGCGAGATCAAGAAGTGGTTCAGTGACCACCGATATCGGTGTCAAAGGGGCATCGTCCACATCACCAGCGAATCCCTTGCCAAAGACCAGTTGGCCATCGCGGCCTCCCGACACGGTCGCACGTATCATGCGTACCCAGACTTTGCCCCCCAGAAGTTCAAAGAGAAAACACAGGGTCAGGTTAAAATCTTGGAAGACAGCTTTTTGAAAAGTTCTTTTCCTACCCAAGCAGAACTGGATCGGCTAAGGGTGGAGACCAAGCTGAGCAGGAGAGAGATCGACTCCTGGTTCTCGGAGAGGCGGAAGCTTCGAGACAGCATGGAACAAGCTGTCTTGGATTCCATGGGGTCTGGCAAAAAAGGCCAAGATGTGGGAGCCCCCAATGGTGCTCTGTCTCGACTCGACCAGCTCTCCGGTGCCCAGTTAACAAGTTCTCTGCCCAGCCCTTCGCCAGCAATTGCAAAAAGTCAAGAACAGGTTCATCTCCTGAGGAGCACGTTTGCAAGAACCCAGTGGCCTACTCCCCAGGAGTACGACCAGTTAGCGGCCAAGACTGGCCTGGTCCGAACTGAGATTGTGCGTTGGTTCAAGGAGAACAGATGCTTGCTGAAAACGGGAACCGTGAAGTGGATGGAGCAGTACCAGCACCAGCCCATGGCAGATGATCACGGCTACGATGCCGTAGCAAGGAAAGCAACAAAACCCATGGCCGAGAGCCCAAAGAACGGGGGTGATGTGGTTCCACAATATTACAAGGACCCCAAAAAGCTCTGCGAAGAGGACTTGGAGAAGTTGGTGACCAGGGTAAAAGTAGGCAGCGAGCCAGCAAAAGACTGTTTGCCAGCAAAGCCCTCAGAGGCCACCTCAGACCGGTCAGAGGGCAGCAGCCGGGACGGCCAGGGTAGCGACGAGAACGAGGAGTCGAGCGTTGTGGATTACGTGGAGGTGACGGTCGGGGAGGAGGATGCCATCTCAGATAGATCAGATAGCTGGAGTCAGGCTGCGGCAGAAGGTGTGTCGGAACTGGCTGAATCAGACTCCGACTGCGTCCCTGCAGAGGCTGGCCAGGCCGGTATGGACTACAAGGATGACGATGACAAGGATTACAAAGACGACGATGATAAGGACTATAAGGATGATGACGACAAATGAGCTAGCCTGTGGAATGTGTGTCAGTTAGGGTGTGGAAAGTCCCCAGGCTCCCCAGCAGGCAGAAGTATGCAAAGCATGCATCTCAATTAGTCAGCAACCAGGTGTGGAAAGTCCCCAGGCTCCCCAGCAGGCAGAAGTATGCAAAGCATGCATCTCAA

The red font is the insertion sequence, the black font is the carrier sequence, and the underline mark is the enzyme cutting site.

**
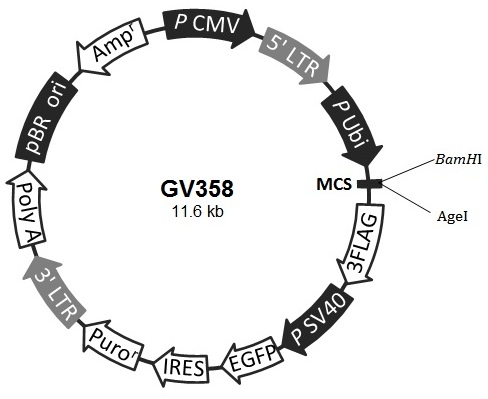
**

**LV-shVHL**

1.PSC75485-1-H1-F_B05.ab1NNGNCNNCAACTAAAGATTACAAAACAAATTACAAAAATTCAAAATTTTCGGGTTTATTACAGGGACAGCAGAGATCCAGTTTGGTTAATTAATCGAGCGGCCGCCCCCTTCACCGAGGGCCTATTTCCCATGATTCCTTCATATTTGCATATACGATACAAGGCTGTTAGAGAGATAATTGGAATTAATTTGACTGTAAACACAAAGATATTAGTACAAAATACGTGACGTAGAAAGTAATAATTTCTTGGGTAGTTTGCAGTTTTAAAATTATGTTTTAAAATGGACTATCATATGCTTACCGTAACTTGAAAGTATTTCGATTTCTTGGCTTTATATATCTTGTGGAAAGGACGAAACACCGGGATCTGGAAGACCACCCAAATCTCGAGATTTGGGTGGTCTTCCAGATCTTTTTGAATTCTCGACCTCGAGACAAATGGCAGTATTCATCCACGGATCCTAACCCGTGTCGGCTCCAGATCTGGCCTCCGCGCCGGGTTTTGGCGCCTCCCGCGGGCGCCCCCCTCCTCACGGCGAGCGCTGCCACGTCAGACGAAGGGCGCAGCGAGCGTCCTGATCCTTCCGCCCGGACGCTCAGGACAGCGGCCCGCTGCTCATAAGACTCGGCCTTAGAACCCCAGTATCAGCAGAAGGACATTTTAGGACGGGACTTGGGTGACTCTAGGGCACTGGTTTTCTTTCCAGAGAGCGGAACAGGCGAGGAAAAGTAGTCCCTTCTCGGCGATTCTGCGGAGGGATCTCCGTGGGGCGGTGAACGCCGATGATTATATAAGGACGCGCCGGGTGTGGCACAGCTAGTTCCGTCGCAGCCGGGATTTGGGTCGCGGTTCTTGTTTGTGGATCGCTGTGATCGTCACTTGGTGAGTAGCGGGCTGCTGGGCTGGCCGGGGCTTTCGTGGCCGCCGGGCCGCTCGGTGGACGGAAGCGTGTGGAGAGACCGCCAAGGGCTGTAGTCTGGGTCCGCGAGCAAGGTTGCCCTGAACTGGGGGTTTGGGGGGGAGCCGCAGCAAAA

2.PSC75486-1-H1-F_C02.ab1NNNCNNNNNNAACTAAAGATTACAAAAACAAATTACAAAAATTCAAAATTTTCGGGTTTATTACAGGGACAGCAGAGATCCAGTTTGGTTAATTAAGATCCGACGCGCCATCTCTAGGCCCGCGCCGGCCCCCTCGCACAGACTTGTGGGAGAAGCTCGGCTACTCCCCTGCCCCGGTTAATTTGCATATAATATTTCCTAGTAACTATAGAGGCTTAATGTGCGATAAAAGACAGATAATCTGTTCTTTTTAATACTAGCTACATTTTACATGATAGGCTTGGATTTCTATAACTTCGTATAGCATACATTATACGAAGTTATAAACAGCACAAAAGGAAACTCACCCTAACTGTAAAGTAATTGTGTGTTTTGAGACTATAAATATCCCTTGGAGAAAAGCCTTGACCGGGTCGAAGAGTACGGCCCTGAACTCGAGTTCAGGGCCGTACTCTTCGACTTTTTGGATCCTAACCCGTGTCGGCTCCAGATCTGGCCTCCGCGCCGGGTTTTGGCGCCTCCCGCGGGCGCCCCCCTCCTCACGGCGAGCGCTGCCACGTCAGACGAAGGGCGCAGCGAGCGTCCTGATCCTTCCGCCCGGACGCTCAGGACAGCGGCCCGCTGCTCATAAGACTCGGCCTTAGAACCCCAGTATCAGCAGAAGGACATTTTAGGACGGGACTTGGGTGACTCTAGGGCACTGGTTTTCTTTCCAGAGAGCGGAACAGGCGAGGAAAAGTAGTCCCTTCTCGGCGATTCTGCGGAGGGATCTCCGTGGGGCGGTGAACGCCGATGATTATATAAGGACGCGCCGGGTGTGGCACAGCTAGTTCCGTCGCAGCCGGGATTTGGGTCGCGGTTCTTGTTTGTGGATCGCTGTGATCGTCACTTGGTGAGTAGCGGGCTGCTGGGCTGGCCGGGGCTTTCGTGCCGCCGGGCCGCTCGGTGGGACGGAAGCCTGTGGAGAGACCGCC

3.PSC75487-1-H1-F_D02.ab1NNNNNNNNANCNANTAAGATTACAAAAACAAATTACAAAAATTCAAAATTTTCGGGTTTATTACAGGGACAGCAGAGATCCAGTTTGGTTAATTAAGATCCGACGCGCCATCTCTAGGCCCGCGCCGGCCCCCTCGCACAGACTTGTGGGAGAAGCTCGGCTACTCCCCTGCCCCGGTTAATTTGCATATAATATTTCCTAGTAACTATAGAGGCTTAATGTGCGATAAAAGACAGATAATCTGTTCTTTTTAATACTAGCTACATTTTACATGATAGGCTTGGATTTCTATAACTTCGTATAGCATACATTATACGAAGTTATAAACAGCACAAAAGGAAACTCACCCTAACTGTAAAGTAATTGTGTGTTTTGAGACTATAAATATCCCTTGGAGAAAAGCCTTGACCGGTCCCAGGTCATCTTCTGCAATCTCGAGATTGCAGAAGATGACCTGGGATTTTTGGATCCTAACCCGTGTCGGCTCCAGATCTGGCCTCCGCGCCGGGTTTTGGCGCCTCCCGCGGGCGCCCCCCTCCTCACGGCGAGCGCTGCCACGTCAGACGAAGGGCGCAGCGAGCGTCCTGATCCTTCCGCCCGGACGCTCAGGACAGCGGCCCGCTGCTCATAAGACTCGGCCTTAGAACCCCAGTATCAGCAGAAGGACATTTTAGGACGGGACTTGGGTGACTCTAGGGCACTGGTTTTCTTTCCAGAGAGCGGAACAGGCGAGGAAAAGTAGTCCCTTCTCGGCGATTCTGCGGAGGGATCTCCGTGGGGCGGTGAACGCCGATGATTATATAAGGACGCGCCGGGTGTGGCACAGCTAGTTCCGTCGCAGCCGGGATTTGGGTCGCGGTTCTTGTTTGTGGATCGCTGTGATCGTCACTTGGTGAGTAGCGGGCTGCTGGGCTGGCCGGGGCTTTCGTGGCCGCCGGGCCGCTCGGTGGGACGGAAGCGTGTGGAGAGACCGCCAAGGGCTGTAGTC


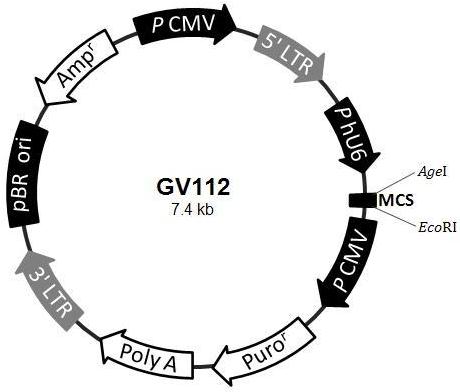


**Table S2**

QPCR Primer sequence

| Gene | primer F(5'-3') | primer R |
| --- | --- | --- |
| ZHX2 | CTGCCTTAGCCCCACAC | TGCTACCCAGTTCTCCCA |
| EPAS1 | TGATGTGGAAACGGATGA | ATGGGGTTTTGGGTGAA |
| EGFR | GCCTGAAAACAGGACGGA | GAGGGAGCGTAATCCCAAG |
| VEGF | CTGGGCTGTTCTCGCTT | CCCCTCTCCTCTTCCTTCT |
| TGFB1 | ACCACACCAGCCCTGTTC | CGTCAGCACCAGTAGCCA |
| CXCR4 | CAAGCAAGGGTGTGAGTTT | AGCATAGAGGATGGGGTTC |
| BNIP3 | GGGCTCCTGGGTAGAACT | CAGACTCATGCTGTGCGT |
| MMP2 | CGCCTTTAACTGGAGCAAA | AGGTTATCGGGGATGGC |
| MMP9 | ACGCAGACATCGTCATCC | CCAGGGACCACAACTCG |
| MAP2K1 | CTGCTGTTCCTGCTCCA | TCACAAGGCTCCCTCCTA |
| ERK1 | GCTGGCTCACCCCTACC | GGAAGCGTGCTGTCTCCT |
| ERK2 | CCAGAGCAAGTCCTCCAG | GGCACCAACAGTACAAAGC |
| ERK3 | TCCGGCTGAGTTTGTGC | CAGAGGGCTTGCATTGGT |
| ERK5 | GGACTTCGGGAGCCAGT | AGCAGGGCCAGGTTCTT |
| JIP1 | GAATGGCGGAGCGAGAA | GGTGAGCCTGAAATTGGGA |
| JIP2 | GCTGGAGCTGGATGTGGA | GGGCGTAGAAGGCAGGAA |
| JIP3 | CTAGGGTTGCCCGCTGT | CTGCTTGGTGGCCTCTCT |
| MAPK15 | GAGGACTGGTGAGGTCGTG | GTGTGTGGGTGGGTGGA |
| MAPKAPK2 | CTGCTCAAGGTCACGCA | GCCAGGAGTCAGGAAACC |
| MAPKAPK3 | CCTTGGGGAGGCTGATT | GGGAATAGTGAGGGGATGG |
| MAP2K7 | CGGGAACAAGGAGGAGAA | GGTGATGAACGTCCCAAAG |

**Table S3** Summary of univariate and multivariate Cox regression analysis of OS duration in all ccRCCs.

| Variables |  | Univariate analysis | | | Multivariate analysis | | |
| --- | --- | --- | --- | --- | --- | --- | --- |
|  |  | HR | (95% CI) | *P*-value | HR | (95% CI) | *P*-value |
| ZHX2 expression |  |  |  |  |  |  |  |
| (Low vs High) |  | 0.934 | 2.544(1.504-4.303) | 0.031 | 0.790 | 2.204(1.186-4.099) | 0.112 |
| Gender |  |  |  |  |  |  |  |
| (Male vs Female) |  | 1.120 | 0.828(0.463-1.480) | 0.524 |  |  |  |
| Age(years) |  |  |  |  |  |  |  |
| (≤55 vs >55) |  | 0.580 | 1.786(1.055-3.022) | 0.031 | 0.773 | 2.167(1.148-4.090) | 0.017 |
| TNM stage |  |  |  |  |  |  |  |
| (I+II vs III+IV) |  | 1.710 | 5.529(3.363-9.088) | <0.001 | 1.246 | 3.476(1.996-6.053) | <0.001 |
| Fuhrman grade |  |  |  |  |  |  |  |
| (I+II vs III+IV) |  | 1.466 | 4.333(2.390-7.856) | <0.001 | 0.815 | 2.259(1.117-4.568) | 0.023 |
| Tumor size(cm) |  |  |  |  |  |  |  |
| (≤4 vs >4) |  | 1.964 | 7.129(3.740-13.588) | <0.001 | 1.140 | 3.128(1.488-6.577) | 0.003 |

HR hazard ratio, 95% CI 95% confidence interval

**Table S4.** Summary of univariate and multivariate Cox regression analysis of RFS in all ccRCCs.

| Variables |  | Univariate analysis | | | Multivariate analysis | | |
| --- | --- | --- | --- | --- | --- | --- | --- |
|  |  | HR | (95% CI) | *P*-value | HR | (95% CI) | *P*-value |
| ZHX2 expression |  |  |  |  |  |  |  |
| (Low vs High) |  | 0.688 | 1.990(1.180=3.357) | 0.010 | 0.567 | 1.594(0.864-2.944) | 0.035 |
| Gender |  |  |  |  |  |  |  |
| (Male vs Female) |  | 1.184 | 0.882(0.497-1.564) | 0.667 |  |  |  |
| Age(years) |  |  |  |  |  |  |  |
| (≤55 vs >55) |  | 0.398 | 1.488(0.887-2.496) | 0.132 |  |  |  |
| TNM stage |  |  |  |  |  |  |  |
| (I+II vs III+IV) |  | 1.686 | 5.397(3.295-8.839) | <0.001 | 1.091 | 2.978(1.801-4.925) | <0.001 |
| Fuhrman grade |  |  |  |  |  |  |  |
| (I+II vs III+IV) |  | 1.257 | 3.515(1.938-6.374) | <0.001 | 0.576 | 1.779(0.882-3.588) | 0.108 |
| Tumor size(cm) |  |  |  |  |  |  |  |
| (≤4 vs >4) |  | 2.101 | 8.173(4.213-15.854) | <0.001 | 1.364 | 3.910(1.868-8.186) | <0.001 |

HR hazard ratio, 95% CI 95% confidence interval
