# Supplementary material for: ZHX2 drives cell growth and migration via activating MEK/ERK signal and induces Sunitinib resistance by regulating the autophagy in clear cell Renal Cell Carcinoma
Source: Cell Death Dis. 2020 May 7;11(5):337. doi: 10.1038/s41419-020-2541-x (PMC7206010; doi:10.1038/s41419-020-2541-x)
Supplement: Supplementary file 2 — supplementary figure legend [file 41419_2020_2541_MOESM2_ESM.docx]

Supplementary Figures

Figure S1

A. The OS analysis of ccRCC patients obtained from TAGA database.

B-C. The representative images of wound healing assay after ZHX2 or EPAS1 knockdown in 786-O cells.

*p<0.05.

Figure S2

A-B. Wound healing assay showed that ZHX2 overexpression increased cell proliferation and migration in 786-O cells.

C. Bar graph of the tumor weight in 786-O/LV-ZHX2 cells and negative control.

D-E. Wound healing assay showed that cell proliferation and migration abilities were increased in reprogrammed CAKI-1 cells.

*p<0.05, **p<0.01

Figure S3

The immunohistochemistry (IHC) analysis was performed by using the slices of the tumor xenografts. The Ki-67 staining showed that the positive rate was much higher in LV-ZHX2 mouse compared with negative control.

Figure S4

The immunofluorescence staining of CD31 in programmed 786-O cells to detect the blood vessel formation ability. anti-ZHX2 in green, anti-CD31 in red and DAPI in blue.

Figure S5

The semi-quantification of the western blot performend in our study. A. The protein level of ERK pathway was detected in reprogrammed ccRCC cell lines. B. The western blot assay performed by mouse tumor tissues have showed the MAPK-related protein were up-regulated in LV-ZHX2 group. The ratio was normalized by GAPDH respectively.

Figure S6

A. Sunitinib inhibition rates were tested in 786-O and CAKI-1 cell lines respectively.

B. Chloroquine inhibition rates were tested in 786-O and CAKI-1 cell lines respectively.

C. Many vesicles were showed up in 786-O/LV-ZHX2 cells after sunitinib treatment.
