# Supplementary figures and images for: ZHX2 drives cell growth and migration via activating MEK/ERK signal and induces Sunitinib resistance by regulating the autophagy in clear cell Renal Cell Carcinoma
Source: Cell Death Dis. 2020 May 7;11(5):337. doi: 10.1038/s41419-020-2541-x (PMC7206010; doi:10.1038/s41419-020-2541-x)

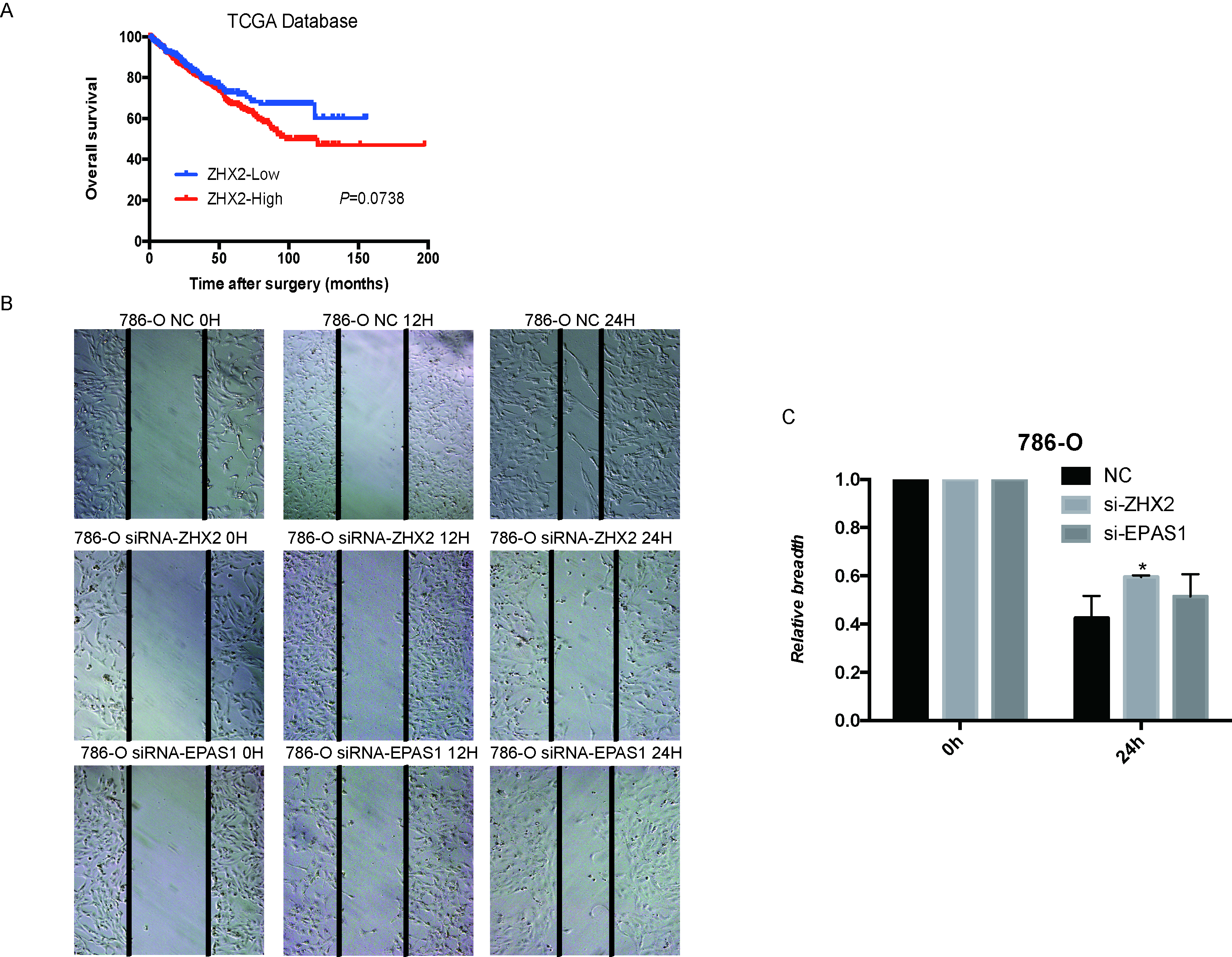

Supplement: Supplementary file 3 — supplementary S1 [file 41419_2020_2541_MOESM3_ESM.tif]

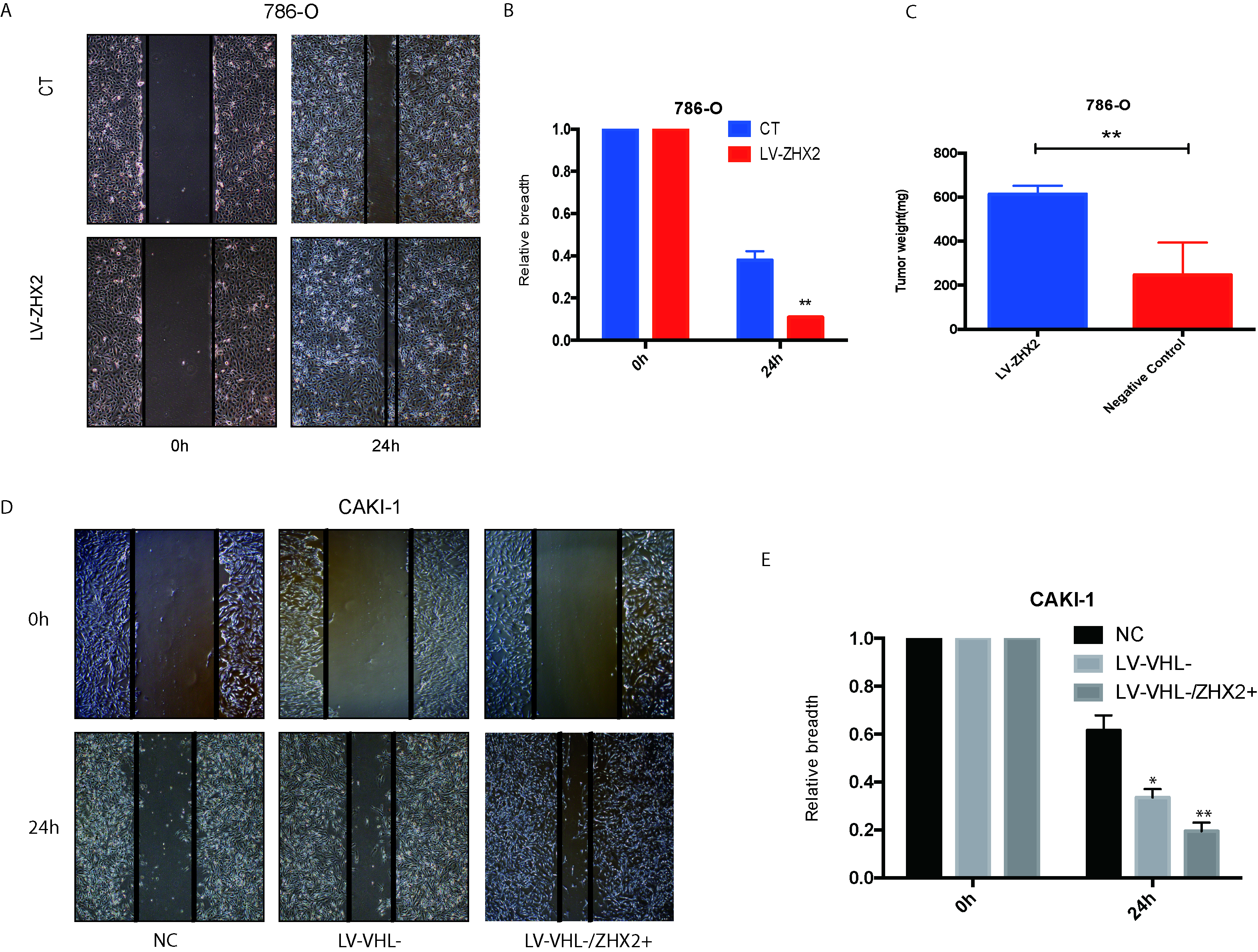

Supplement: Supplementary file 4 — supplementary S2 [file 41419_2020_2541_MOESM4_ESM.tif]

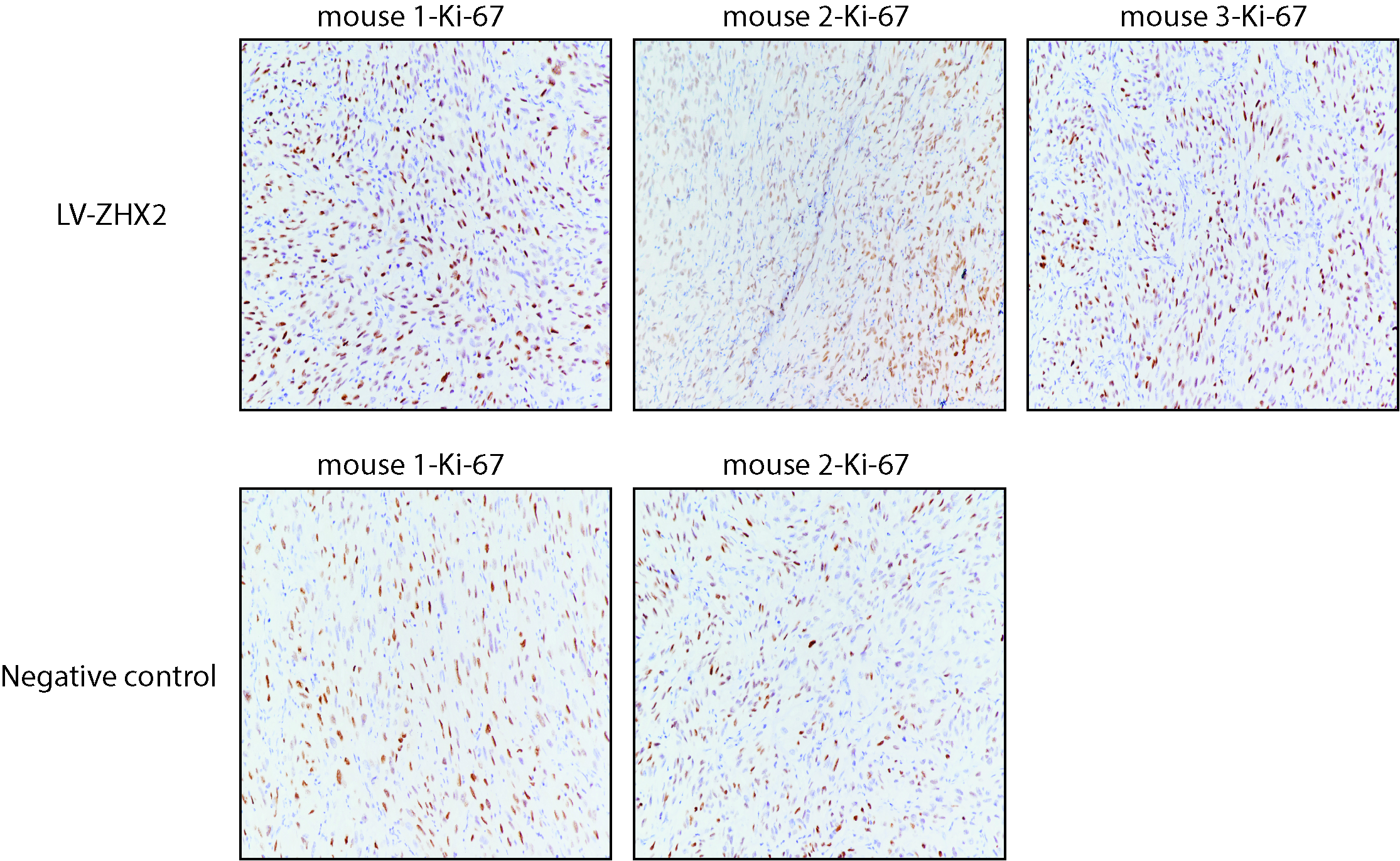

Supplement: Supplementary file 5 — supplementary S3 [file 41419_2020_2541_MOESM5_ESM.tif]

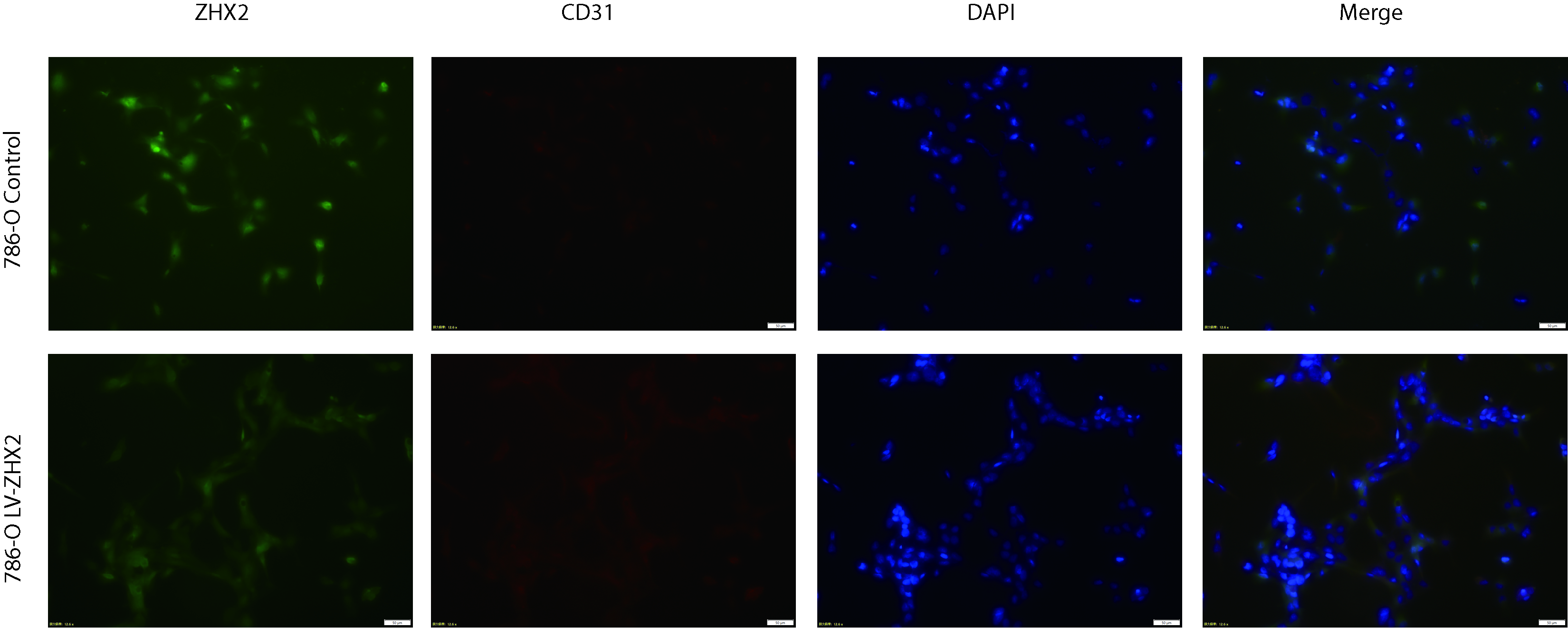

Supplement: Supplementary file 6 — supplementary S4 [file 41419_2020_2541_MOESM6_ESM.tif]

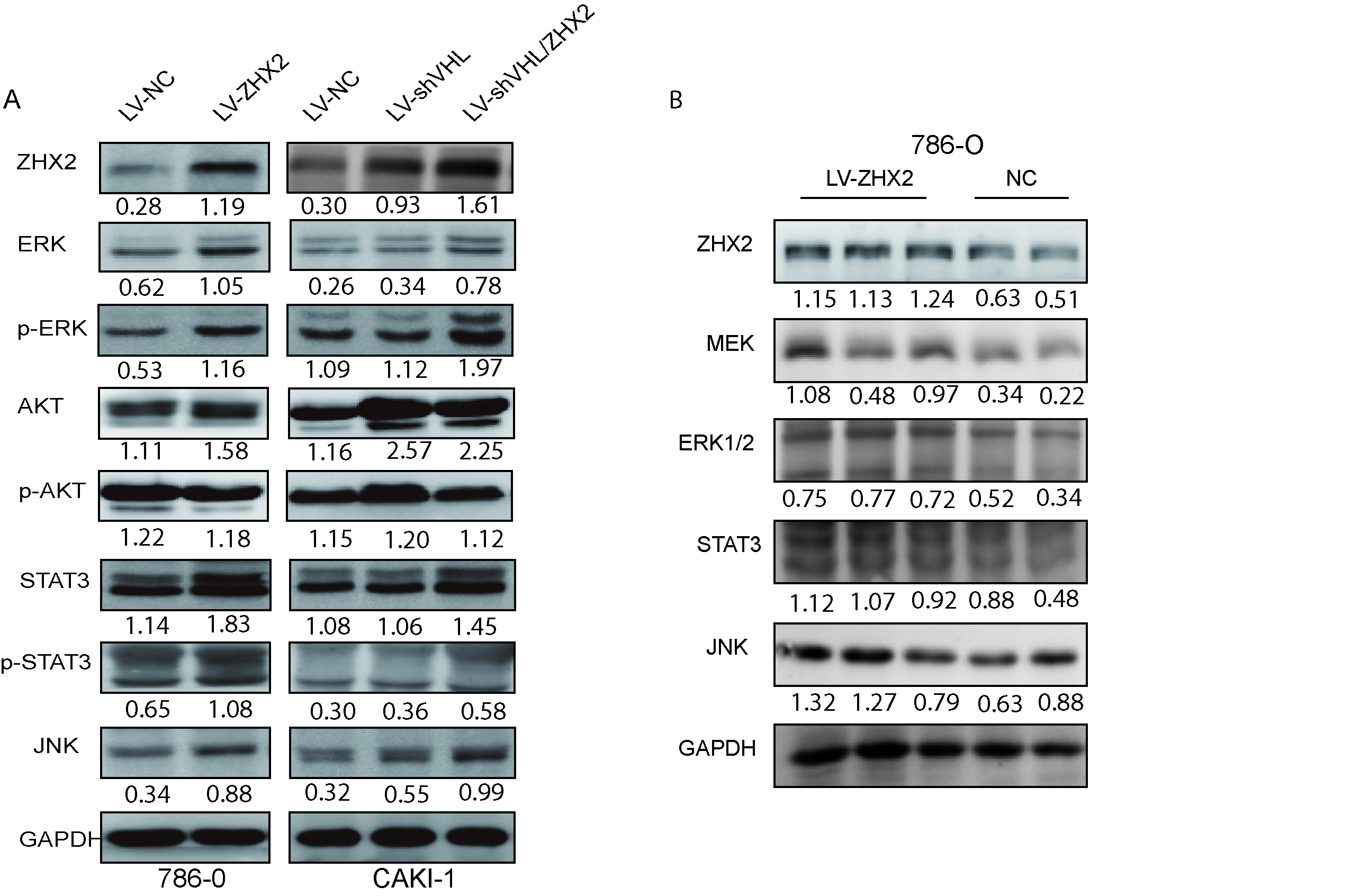

Supplement: Supplementary file 7 — supplementary S5 [file 41419_2020_2541_MOESM7_ESM.tif]

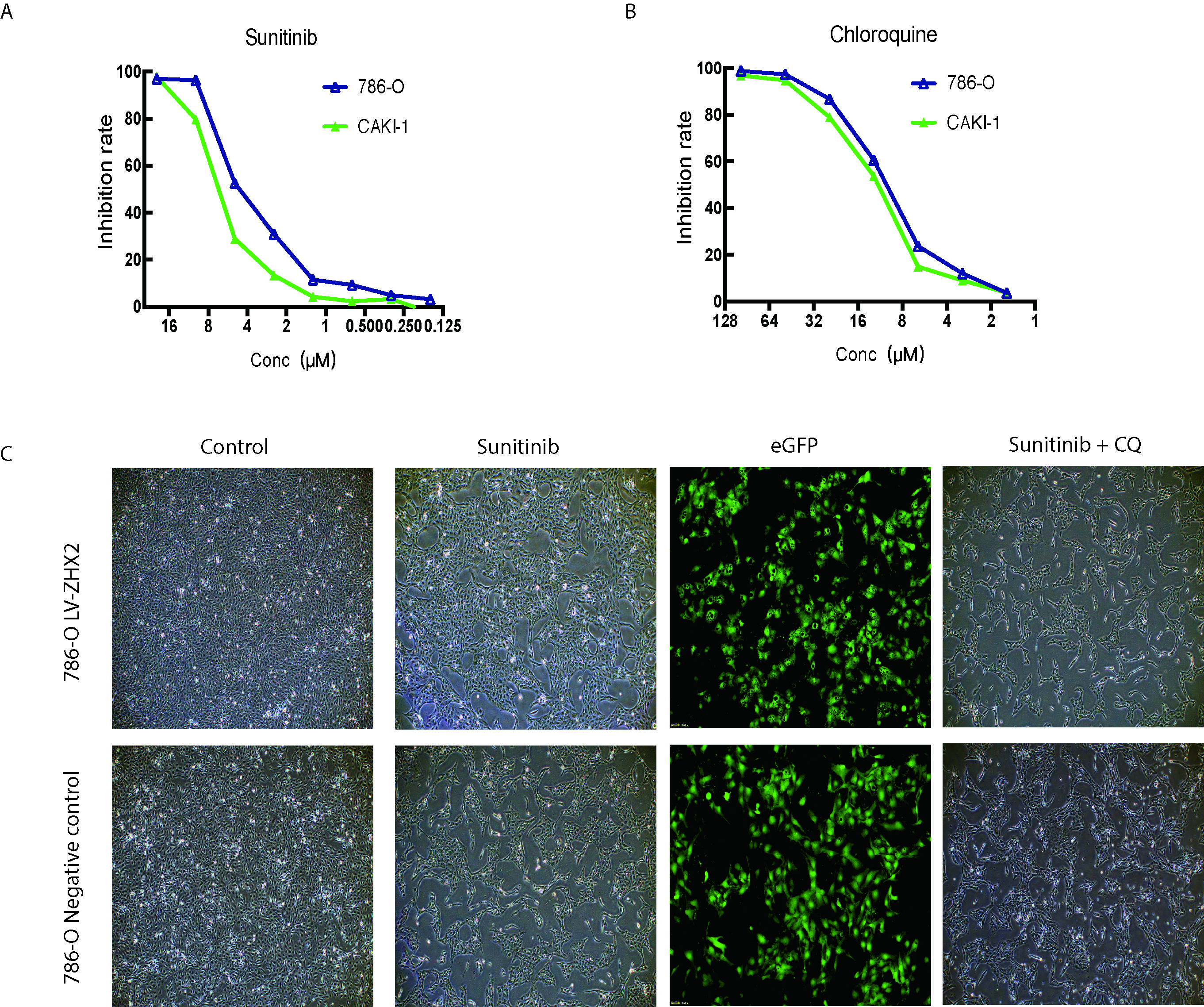

Supplement: Supplementary file 8 — supplementary S6 [file 41419_2020_2541_MOESM8_ESM.tif]
